# RBM47 restrains renal cell carcinoma progression and chemoresistance through interacting with lncRNA HOXB-AS1

**DOI:** 10.1038/s41420-023-01623-7

**Published:** 2023-09-02

**Authors:** Qingfu Su, Zhenliang Pan, Heyi Chen, Jiabi Chen, Yanmei Zhang, Wei Zhuang

**Affiliations:** Department of Urology, The Second Affiliated Hospital of Fujian Medical University, Quanzhou Fujian, China

**Keywords:** Renal cancer, Epigenetics

## Abstract

RNA binding proteins have the critical role in renal cell carcinoma (RCC) progression. However, the role of RBM47 in RCC has not been elucidated. In this study, we found that RBM47 was downregulated in RCC tissues and its expression was negatively correlated with the prognosis of RCC patients. Also, we found that the expression of RBM47 was regulated by CBP/P300-mediated H3K27ac in RCC. Functionally, RBM47 restrained RCC cells proliferation and metastasis. Mechanistically, RBM47 interfered with the interaction between HOXB-AS1 and p53 proteins via directly binding with HOXB-AS1, finally promoted the entry of p53 into the nucleus and therefore activated the p53 signaling. Moreover, RBM47 had a synergistic anticancer effect with sunitinib both in vivo and in vitro.

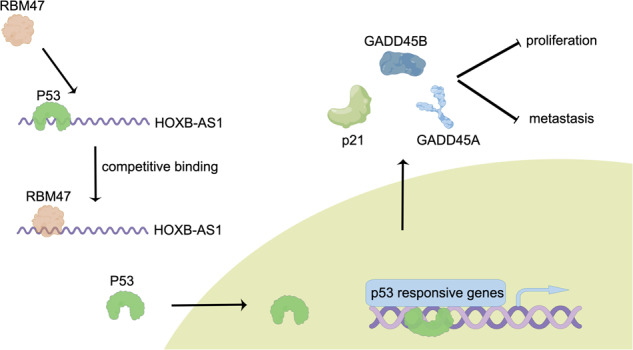

## Introduction

Renal cell carcinoma (RCC) is one of the most lethal types of cancer among urological malignancies [1]. More than 10% of RCC patients are diagnosed with distant metastases, and the 5-year survival rate of metastasized RCC patients is only 12% [2]. Although the prognosis of RCC patients has been improved through targeted therapy, including tyrosine kinase inhibitors and rapamycin inhibitors, most of them are prone to be drug resistance, and the survival period of these patients is still not optimistic [3–6]. Therefore, it is urgent to explore the new therapeutic strategies for RCC.

RNA-binding motif (RBM) proteins are a subgroup of RNA binding proteins that contain RNA-binding domains and RNA reorganization motifs [7]. RBMs are widely involved in various physiological and pathological processes, including RNA transcription, pre-mRNA splicing, translocation, and RNA stability [8]. Recently, various RBMs were reported to be involved in cancer progression. For instance, RBM38 could destabilize MDM2 mRNA and restore p53 expression to inhibit hepatocellular carcinoma progression [9]. RBM10 restrained lung cancer metastasis through regulating the alternative splicing of lncRNA Neat1 [10]. However, the role of RBM47 in carcinogenesis is still unclear. RBM47 could restrain the progression of papillary thyroid carcinoma, hepatocellular carcinoma, and lung adenocarcinoma, while promoting nasopharyngeal progression [11–14]. Therefore, it is meaningful to explore the role of RBM47 in clear cell carcinoma.

Long noncoding RNAs (lncRNAs) has been reported as key regulators in cancer development, especially in renal cell carcinoma [15, 16]. LncRNAs can interact with DNA, RNA, and proteins to regulate target gene expression at the epigenetic, transcriptional or post-transcriptional level [17–19]. For example, lncRNA ADIRF-AS1 could interact with PBAF and regulate downstream genes, such as CCND1 and VEGFA expression to promote renal cell carcinoma tumorigenesis [20]. HOXB-AS1 is an oncogenic lncRNA which accelerates the progression of glioblastoma, multiple myeloma, and endometrial carcinoma [21–24]. However, the role of HOXB-AS1 in renal cell carcinoma is still unclear so far.

In this study, we identified that RBM47 was downregulated in RCC specimens and this was negatively correlated with the prognosis of RCC patients. RBM47 restrained RCC cells proliferation and metastasis in vivo and in vitro. RBM47 could destroy the interaction between lncRNA HOXB-AS1 and p53 protein, promoting the entry of p53 into the nucleus and therefore activating p53 signaling. Moreover, the overexpression of RBM47 and sunitinib had a synergistic anticancer effect on renal cell carcinoma cells.

## Results

### RBM47 was downregulated in RCC

**Fig. 1:**
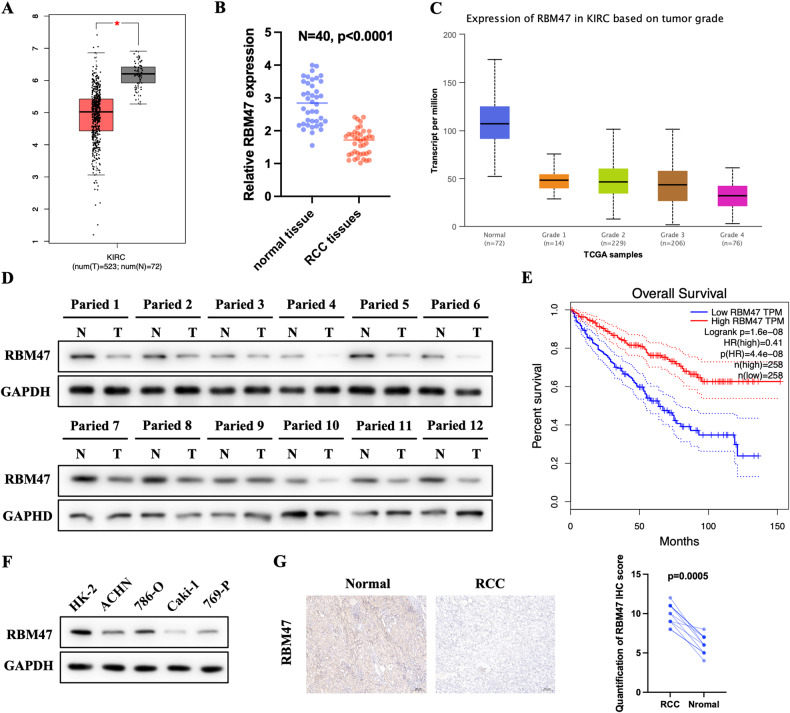
RBM47 was downregulated in RCC. **A** Expression of RBM47 in KIRC patients based on the TCGA database using the GEPIA2 platform. **B** Relative mRNA level of RBM47 measured by qRT-PCR in our 40 paired RCC patients’ specimens. **C** Expression of RBM47 in different stages of KIRC patients based on the TCGA database using the UALCAN platform. **D** Relative protein level of RBM47 measured by western blot in our 12 paired RCC patients’ specimens. **E** Overall KIRC patients’ survival determined by RBM47 expression using the GEPIA2 platform. **F** Relative protein level of RBM47 measured by western blot in RCC cell lines and normal cell lines. **G** Representative IHC images and IHC scores in 12 paired RCC patients’ specimens. Data are presented as mean ± SD in three independent experiments. *p < 0.05.

In order to validate the expression pattern of RBM47 in renal cell carcinoma, we first evaluate the expression of RBM47 in the TCGA-KIRC database. The result showed that RBM47 was downregulated in renal cell carcinoma tissues compared with normal tissues (Fig. 1A). Then, the qRT-PCR result of 40 paired RCC and corresponding normal specimens indicated that RBM47 was downregulated in RCC tissues (Fig. 1B). Also, the expression of RBM47 was negatively correlated with the tumor grade of RCC according to the TCGA-KIRC database (Fig. 1C). Then, we evaluated the protein expression of RBM47 in our 12 paired RCC and adjacent normal tissues. The result proved that RBM47 was lowly expressed in RCC (Fig. 1D). Survival analysis also demonstrated that high expression of RBM47 was associated with good overall survival and longer disease-free survival period (Fig. 1E and Supplementary Fig. 1A). Next, we demonstrated that the expression of RBM47 was downregulated in RCC cell lines (ACHN, 786-O, Caki-1, and 769-P) compared with the normal renal cell line (HK-2) (Fig. 1F). Finally, we measured the expression RBM47 in RCC specimens using IHC, and the results demonstrated that RBM47 was downregulated in RCC tissues compared with normal tissues (Fig. 1G). Altogether, these clinical data indicated that RBM47 was downregulated in RCC.

### RBM47 was regulated by CBP/P300-mediated H3K27ac in RCC

**Fig. 2:**
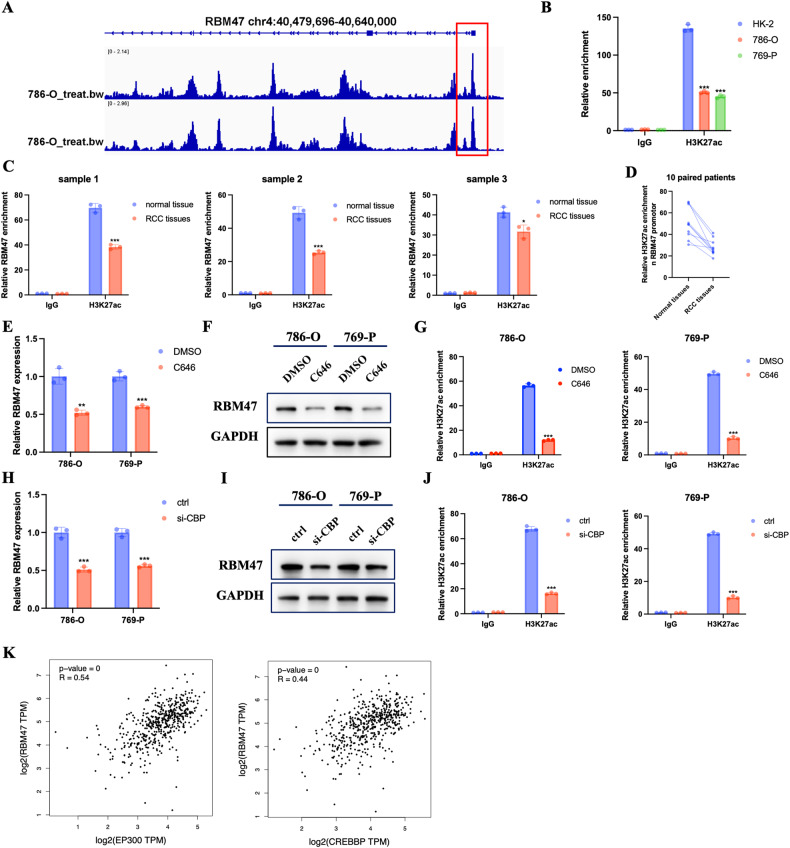
RBM47 was regulated by CBP/P300-mediated H3K27ac in RCC. **A** IGV showing the H3K27ac modification in RBM47 gene promotor using the Cistrome database. **B** Relative H3K27ac modification of RBM47 promotor in 786-O, 769-P and HK-2 cell lines using CHIP-qPCR. **C** Relative H3K27ac modification of RBM47 promotor in 3 RCC patients using CHIP-qPCR. **D** Representative H3K27ac modification of RBM47 promotor in 10 paired RCC patients’ specimens. **E**, **F** Relative mRNA and protein expression of RBM47 in RCC cells treated with C646. **G** Relative H3K27ac modification of RBM47 promotor in RCC cells treated with C646 using CHIP-qPCR. **H**, **I** Relative mRNA and protein expression of RBM47 in CBP knockdown RCC cells. **J** Relative H3K27ac modification of RBM47 promotor in CBP knockdown RCC cells using CHIP-qPCR. **K** Correlation analysis between RBM47 and P300 or CBP using the TCGA-KIRC database. Data are presented as mean ± SD in three independent experiments. *p < 0.05;**p < 0.01; ***p < 0.001.

To investigate the reason why RBM47 was downregulated in RCC cells, we searched the UCSC (http://genome-asia.ucsc.edu/). Surprisingly, we found that the promotor region of RBM47 was enriched with H3 lysine 27 acetylation (H3K27ac) modification (Supplementary Fig. 1B). Also, Cistrome database (http://cistrome.org/db/#/) also confirmed the enrichment of H3K27ac modification in the RBM47 promotor of 786-O cells (Fig. 2A). Thus, we hypothesized that RBM47 was regulated by H3K27ac modification. Subsequently, CHIP-qPCR assays were conducted to confirm this hypothesis. The results demonstrated that the H3K27ac modification was higher in HK-2 cells compared with RCC cells (786-O and 769-O) (Fig. 2B). We also compared the H3K27ac modification in 10 paired patients’ tissues. As expected, the H3K27ac modification was downregulated in RCC tissues compared with normal tissues (Fig. 2C, D). The H3K27ac modification, which is normally regulated by histone acetyltransferases CBP and P300, could promote target gene activation [25, 26]. To verify the role of H3K27ac in RBM47 expression, C646, a specific histone acetyltransferase P300 inhibitor, was applied. The results demonstrated that the mRNA and protein expression of RBM47 were decreased upon treatment with C646 (Fig. 2E, F). And the H3K27ac levels in the RBM47 promotor were inhibited in 786-O and 769-P cells when treated with C646 (Fig. 2G). The overexpression of P300 could increase the expression of RBM47 and the enrichment of H3K27ac in the RBM47 promoter of RCC cells (Supplementary Fig. 1C-1E). Next, we knocked down the expression of the other histone acetyltransferase, CBP. The results showed that silencing of CBP decreased the expression of RBM47 in mRNA and protein levels (Fig. 2H, I). And the H3K27ac levels in the RBM47 promotor were inhibited in RCC cells under the knockdown of CBP (Fig. 2J). Also, overexpression of CBP could increase the expression of RBM47 and the enrichment of H3K27ac in the RBM47 promoter of RCC cells (Supplementary Fig. 1F–H). Furthermore, correlation analysis was performed between RBM47 and histone acetyltransferases in the TCGA database. The results indicated that RBM47 was positively correlated with P300 and CBP in the TCGA-KIRC database (Fig. 2K). Totally, these data revealed that RBM47 was regulated by CBP/P300-mediated H3K27ac in RCC.

### RBM47 inhibited RCC progression in vitro

**Fig. 3:**
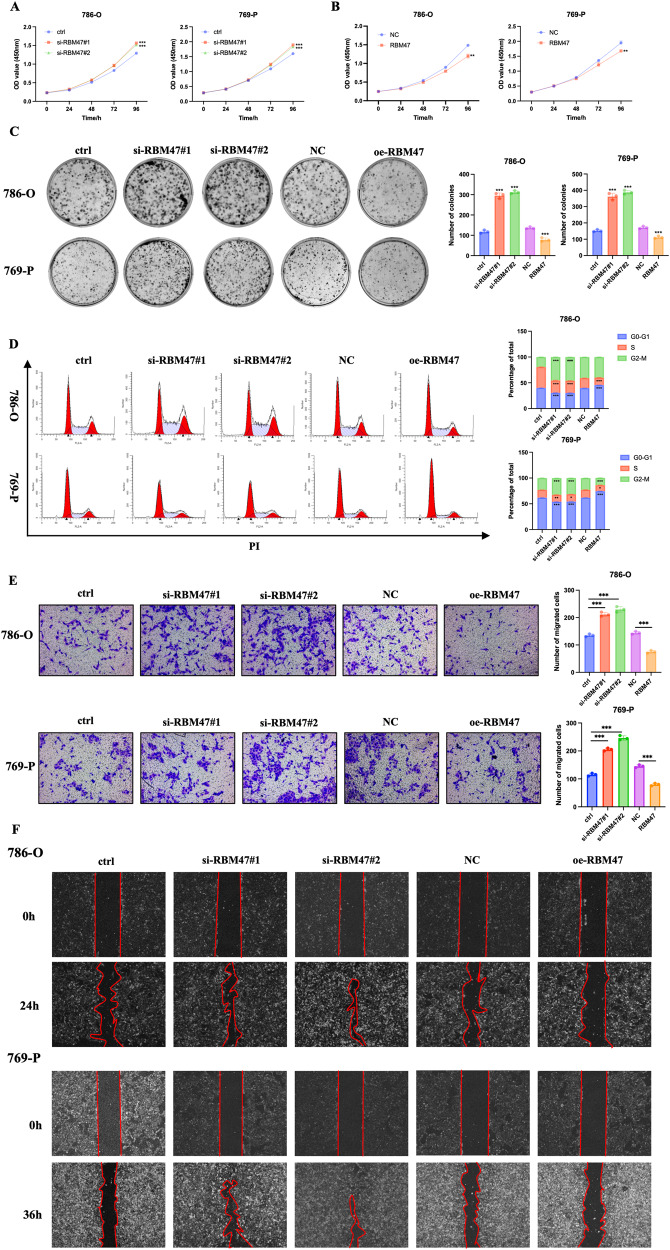
RBM47 inhibited RCC progression in vitro. **A**, **B** CCK-8 assays showing the growth curve of RCC cells upon RBM47 knockdown or overexpression respectively. **C** Colony formation assays showing the growth ability of RCC cells upon RBM47 knockdown or overexpression, respectively. **D** Flow cytometry analysis of cell cycle in RCC cells upon RBM47 knockdown or overexpression. **E** Transwell migration assays showing the metastatic ability of RCC cells upon RBM47 knockdown or overexpression. **F** Wound healing assays showing the metastatic ability of RCC cells upon RBM47 knockdown or overexpression. Data are presented as mean ± SD in three independent experiments. **p < 0.01; ***p < 0.001.

Considering that RBM47 is dysregulated in RCC tissues, we hypothesized whether RBM47 has the vital role in RCC progression. RBM47-specific siRNAs and overexpression plasmids were applied, and the knockdown or overexpression efficiency were examined (Supplementary Fig. 2A, B). CCK-8 assays showed that silencing of RBM47 promoted RCC cells proliferation (Fig. 3A), while overexpression of RBM47 restrained RCC cells proliferation (Fig. 3B). Also, colony formation assays showed similar results (Fig. 3C). Then, flow cytometry assays revealed that silencing of RBM47 decreased the percentage of G0-G1 phase cells, while overexpression of RBM47 lead to the arrest of RCC cells in G0-G1 phase (Fig. 3D). Subsequently, we explored the role of RBM47 in RCC cells’ migration. Transwell assays and wound healing assays were applied to assess the role of RBM47 in RCC migration. The results demonstrated that knockdown of RBM47 promoted RCC cells migration (Fig. 3E), while overexpression of RBM47 inhibited RCC cells migration (Fig. 3F). Altogether, these data revealed that RBM47 restrained RCC progression in vitro.

### RBM47 inhibited RCC cells proliferation in vivo

**Fig. 4:**
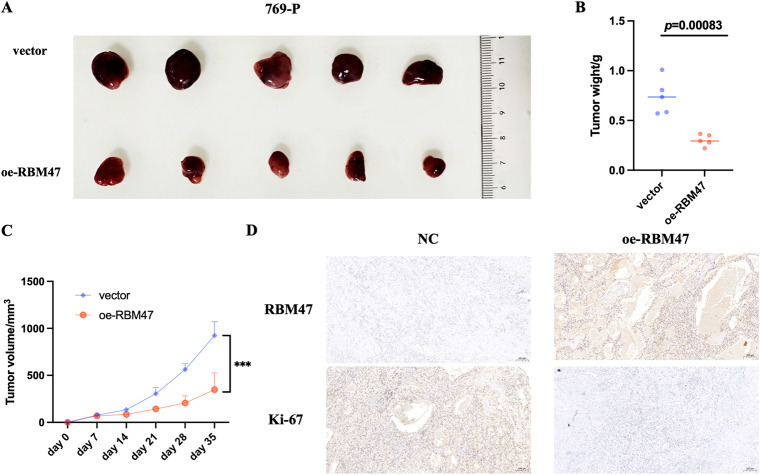
RBM47 inhibited RCC cells proliferation in vivo. **A**–**C** Tumor xenograft model showing the proliferation ability of 769-P cells with or without RBM47 overexpression in nude mice. **D** Representative IHC images in xenograft from vector and oe-RBM47 group. Data are presented as mean ± SD in three independent experiments.***p < 0.001.

To explore the role of RBM47 in RCC proliferation in vivo, RBM47 stably-overexpressed 769-P cells were constructed. Then, RBM47 overexpression or NC 769-P cells were subcutaneously injected into the nude mice. The results showed that RBM47 overexpression significantly reduced the tumor volume and tumor weight (Fig. 4A–C). IHC staining indicated that Ki-67 expression was reduced in RBM47 overexpression tumors (Fig. 4D). Taken together, these data indicated that RBM47 restrained RCC cells proliferation in vivo.

### RBM47 interacted with lncRNA HOXB-AS1 in RCC

**Fig. 5:**
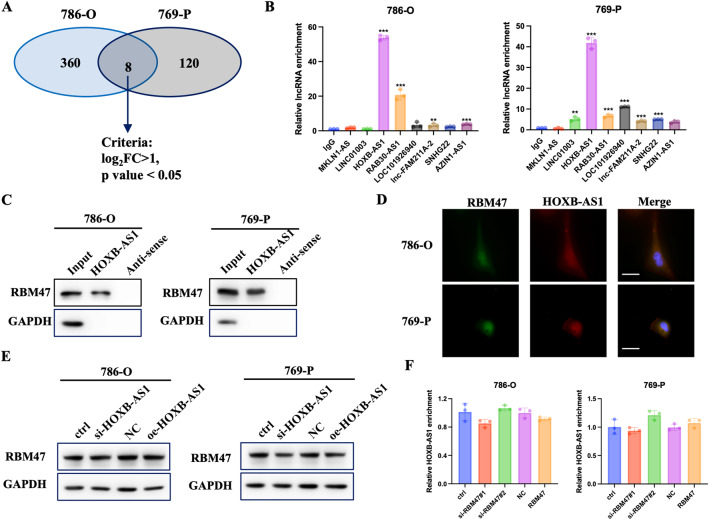
RBM47 interacted with lncRNA HOXB-AS1 in RCC. **A** Venn diagram showing the candidate 8 RBM47-binding lncRNAs using RIP-seq. **B** RIP-qPCR assays showing the binding ability of 8 lncRNAs with RBM47. **C** RNA pulldown assays showing the binding ability of RBM47 with 8 lncRNAs. **D** FISH-IF showing the binding ability of RBM47 with 8 lncRNAs; Bar = 20 µm. **E** The protein level of RBM47 in RCC cells upon HOXB-AS1 knockdown and overexpression. **F** The relative expression of HOXB-AS1 in RCC cells upon RBM47 knockdown and overexpression. Data are presented as mean ± SD in three independent experiments. **p < 0.01; ***p < 0.001.

Previous studies have revealed that RBM family proteins could regulate mRNA stability, influence mRNA alternative splicing and regulate gene transcription [10, 27–30]. However, few studies have demonstrated that RBM47 could interact with lncRNA to regulate cancer progression. Thus, we aimed to explore whether RBM47 could restrain RCC progression by regulating lncRNAs. To identify the RBM47-regulated lncRNAs, RBM47-RIP-seq were performed using 786-O and 769-P cells. A total of 360 RBM47-biniding lncRNAs in 786-O and 120 RBM47-binding lncRNAs in 769-P cells were selected based on the following criteria: (1) log_2_FC > 1; (2) *p* value < 0.05. After combining the 786-O and 769-P sequencing data, 8 lncRNAs (MKLN1-AS, LINC01003, HOXB-AS1, RAB30-AS1, LOC101926940, lnc-FAM211A-2, SNHG22, and AZIN1-AS1) were selected (Fig. 5A). Then, RIP assays demonstrated that RBM47 could interact with HOXB-AS1 (Fig. 5B). RNA pulldown assays and FISH-IF assays also confirmed the interaction between RBM47 and HOXB-AS1 (Fig. 5C, D). Furthermore, we tested the effect of RBM47/HOXB-AS1 complex on one another. Western blot assay demonstrated that HOXB-AS1 didn’t change the expression of RBM47 (Fig. 5E). qRT-PCR assays showed that RBM47 had little effect on HOXB-AS1 expression (Fig. 5F). Totally, these data revealed that RBM47 interacted with lncRNA HOXB-AS1.

### RBM47 promoted p53 nuclear entry through inhibiting the interaction between HOXB-AS1 and p53

**Fig. 6:**
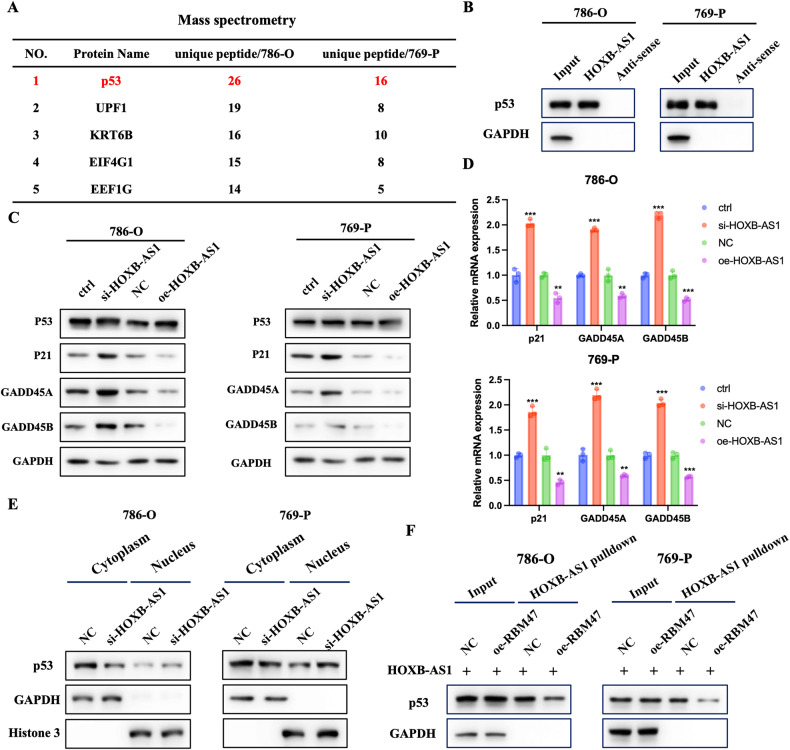
RBM47 promoted p53 nuclear entry through inhibiting the interaction between HOXB-AS1 and p53. **A** The top 5 HOXB-AS1 binding proteins shown in the table using mass spectrometry. **B** RNA pulldown assays showing the binding ability of HOXB-AS1 to p53. **C** The protein expression of p53, p21, GADD45A, and GADD45B in RCC cells upon HOXB-AS1 knockdown or overexpression. **D** The relative mRNA expression of p53, p21, GADD45A, and GADD45B in RCC cells upon HOXB-AS1 knockdown or overexpression. **E** The subcellular distribution of p53 in RCC cells upon HOXB-AS1 knockdown. **F** RNA pulldown assay showing the binding ability of HOXB-AS1 and p53 in RCC cells upon RBM47 overexpression. Data are presented as mean ± SD in three independent experiments. **p < 0.01; ***p < 0.001.

To investigated the detailed mechanism of how RBM47 regulated HOXB-AS1. Considering that RBM47 did not change the expression of HOXB-AS1, we wondered if RBM47 could interfere with the HOXB-AS1 binding capacity in other combinations. Thus, a HOXB-AS1 sense probe was applied to perform the RNA pulldown assay followed by mass spectrometry. A total of 25 proteins, including RBM47, were identified. Among these proteins, p53 was identified to be reproducibly enriched in 786-O and 769-P cells (Fig. 6A). RNA pulldown assay and RIP assay further confirmed the interaction between HOXB-AS1 and p53 (Fig. 6B and Supplementary Fig. 3A). qRT-PCR assay showed that p53 had little effect on HOXB-AS1 expression (Supplementary Fig. 3B). Surprisingly, although HOXB-AS1 didn’t change the expression of p53, HOXB-AS1 negatively regulated the expression of p53 downstream targets, including p21, GADD45A, and GADD45B (Fig. 6C). qRT-PCR results also confirmed this founding (Fig. 6D). These results suggested that HOXB-AS1 could regulate p53 target genes at the transcriptional level. Considering previous studies have reported that lncRNAs could exert their function through altering the subcellular distribution of target proteins [31, 32], we, thus, hypothesized that whether HOXB-AS1 could change the subcellular distribution of p53 to regulate p53 downstream genes expression. Protein subcellular fractionation assays indicated that silencing of HOXB-AS1 increased the nuclear distribution of p53 (Fig. 6E), suggesting that the interaction between HOXB-AS1 and p53 inhibited the entry of p53 into the nucleus. Then, we explored the role of RBM47 in the interaction between HOXB-AS1 and p53. RNA pulldown assays suggested that overexpression of RBM47 could impair the interaction between HOXB-AS1 and p53 (Fig. 6F). Altogether, these data demonstrated that RBM47 could promote the entry of p53 into the nucleus through impairing the interaction between HOXB-AS1 and p53.

### RBM47 restrained RCC progression and activated p53 signaling through HOXB-AS1

**Fig. 7:**
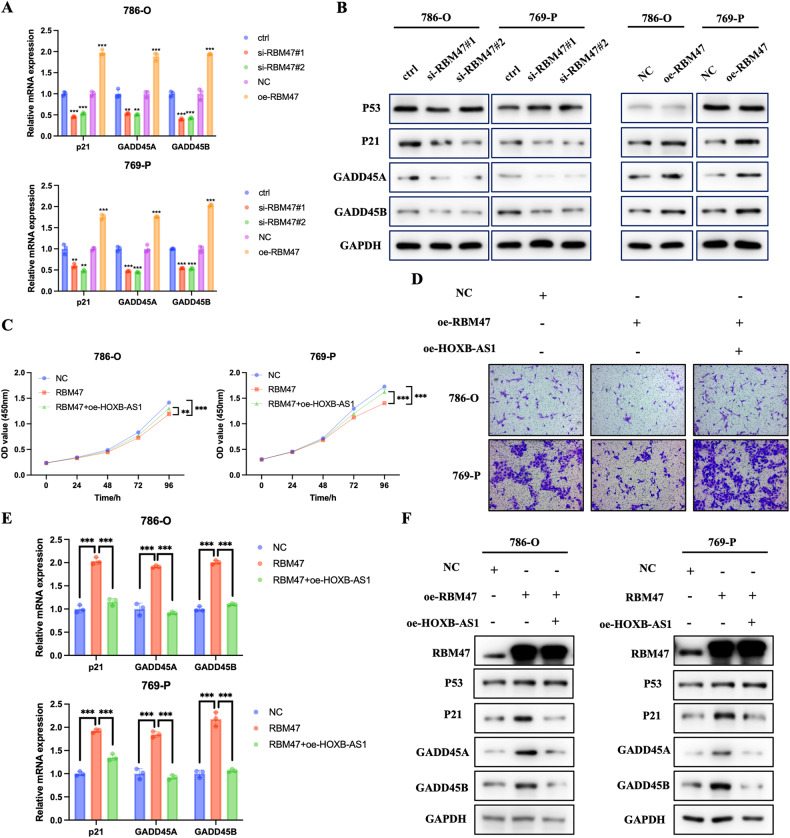
RBM47 restrained RCC progression and activated p53 signaling through HOXB-AS1. **A** The mRNA expression of p21, GADD45A, and GADD45B in RCC cells upon RBM47 knockdown or overexpression. **B** The protein expression of p53, p21, GADD45A, and GADD45B in RCC cells upon RBM47 knockdown or overexpression. **C** CCK-8 assays showing the growth curve of RCC cells upon RBM47 overexpression with or without HOXB-AS1 overexpression. **D** Transwell migration assays showing the metastatic ability of RCC cells upon RBM47 overexpression with or without HOXB-AS1 overexpression. **E** The mRNA expression of p21, GADD45A, and GADD45B in RCC cells upon RBM47 overexpression with or without HOXB-AS1 overexpression. **F** The protein expression of p53, p21, GADD45A, and GADD45B in RCC cells upon RBM47 overexpression with or without HOXB-AS1 overexpression. Data are presented as mean ± SD in three independent experiments. **p < 0.01; ***p < 0.001.

Considering that HOXB-AS1 could inhibit p53 nuclear entry to restrain p53 signaling, and that RBM47 impaired the interaction between HOXB-AS1 and p53, we firstly examined whether RBM47 could activate p53 signaling. qRT-PCR assays showed that silencing of RBM47 inhibited the activation of p53 signaling, while overexpression of RBM47 promoted the activation of p53 signaling (Fig. 7A). Also, western blot assays demonstrated that RBM47 enhanced the expression of p53 downstream genes (Fig. 7B). Furthermore, we explored whether RBM47 promoted RCC progression via binding with HOXB-AS1. CCK-8 assays indicated that overexpression of RBM47 inhibited RCC cells proliferation, while overexpression of HOXB-AS1 could reverse this effect (Fig. 7C). Transwell migration assays demonstrated that overexpression of RBM47 restrained RCC cells migration ability, while overexpression of HOXB-AS1 could reverse this effect (Fig. 7D and Supplementary Fig. 4A). qRT-PCR and western blot assays also demonstrated that overexpression of RBM47 activated the p53 signaling, while overexpression of HOXB-AS1 could reverse this effect (Fig. 7E, F). Collectively, these data indicated that RBM47 inhibited RCC progression and activated p53 signaling through HOXB-AS1.

### RBM47 exerted a synergistic anticancer effect with sunitinib

**Fig. 8:**
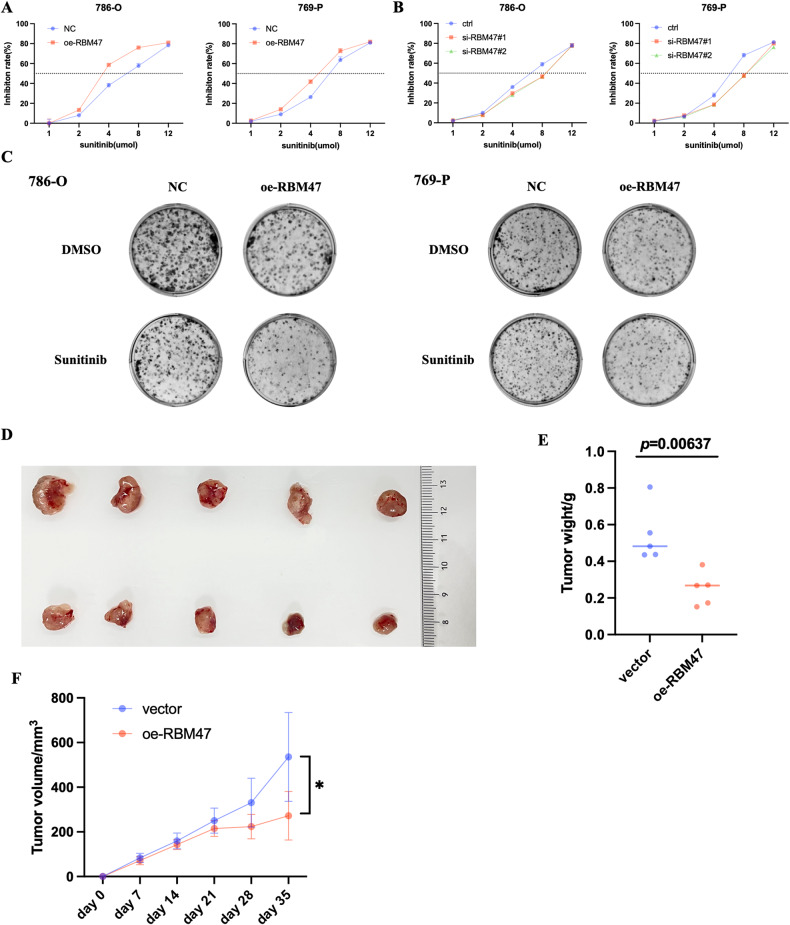
RBM47 exerted synergistic anticancer effect with sunitinib. **A, B** CCK-8 assay of 786-O and 769-P cells with RBM47 knockdown or overexpression treated with sunitinib at the indicated concentration for 48 h. **C** Colony formation assay of 786-O and 769-P cells with sunitinib treatment (1 µm) for 2 weeks. **D**–**F** Tumor xenograft model showing the proliferation ability of 769-P cells with RBM47 overexpression under sunitinib treatment (40 mg/kg/day) for 20 days in nude mice. Data are presented as mean ± SD in three independent experiments. *p < 0.05.

Previous studies have shown that p53 could increase RCC cells sensitivity to sunitinib [33–35]. In our study, we found that RBM47 could restrain RCC cell progression by activating p53 signaling. Thus, we explored whether RBM47 contributed to the chemoresistance of RCC cells. We found that overexpression of RBM47 decreased the IC50 of sunitinib in RCC cells (Fig. 8A), while silencing of RBM47 increased the IC50 of sunitinib in RCC cells (Fig. 8B). Additionally, colony formation assays showed that RBM47 overexpression decreased the resistance of RCC cells to sunitinib (Fig. 8C). Finally, mouse xenograft model demonstrated that overexpression of RBM47 significantly restrained the xenograft growth under sunitinib treatment (40 mg/kg/day) (Fig. 8D–F). Totally, these data demonstrate that RBM47 exerted synergistic anticancer effect with sunitinib.

## Discussion

RBM proteins have been reported to participate in RNA metabolism processes, including pre-mRNA splicing, RNA translocation, and RNA stability [7]. RBM47 is dysregulated in various cancers and is involved in cancer progression [36]. For instance, RBM47 regulated TJP1 alternative splicing to promote lung cancer EMT transition [29]. However, the role of RBM47 in RCC is still unclear. In this study, RBM47 was found to be downregulated in RCC tissues and RCC cell lines. In addition, elevated RBM47 expression was correlated with good prognosis for RCC patients. Functional assays confirmed that RBM47 restrained RCC cells proliferation and metastasis.

H3K27ac is a common histone modification which positively regulates target genes expression [25]. CBP and its related protein P300 are considered to be required for global histone acetylation [37, 38]. In this study, by searching the UCSC and Cistrome databases, we found that the promotor region of RBM47 was enriched with the H3K27ac modification. CHIP-qPCR assays verified the enrichment of H3K27ac modification in the RBM47 promotor region. Also, the H3K27ac modification was downregulated in RCC tissues compared with normal tissues, which suggested the reason why RBM47 was downregulated in RCC tissues. Furthermore, a p300 inhibitor or silencing of CBP decreased the expression of RBM47 in RCC cells.

RBM47 is an RNA binding protein which contains three RNA-recognition motifs (RRMs). Previous studies have reported that RBM47 could regulate RNA transcription, pre-mRNA splicing, translocation, and RNA stability. For example, RBM47 could enhance the IFNAR1 mRNA stability to potentiate host antiviral activity [30]. In addition, RBM47 could recognize the pre-mRNA of TJP1 and regulate its alternative splicing [29]. In this study, RIP-seq was applied to explore RBM47-binding lncRNAs, and HOXB-AS1 was identified to bind with RBM47. RNA pulldown and RIP-qPCR assays also confirmed the sequencing data. Considering that RBM47 has three RRMs, we speculated that RBM47 bind to HOXB-AS1 through its RRMs. HOXB-AS1 was an oncogenic lncRNA which promoted glioblastoma and multiple myeloma progression [21, 22]. However, the role of HOXB-AS1 in renal cell carcinoma has not been discussed. In our study, we found that the binding of RBM47 and HOXB-AS1 didn’t alter the respective expression of RBM47 or HOXB-AS1. To explore how RBM47 regulated HOXB-AS1, lncRNA pulldown followed by mass spectrometry were performed, and p53 was selected. RNA pulldown and RIP-qPCR assays also confirmed the interaction of HOXB-AS1 and p53. Previous studies demonstrated that the C terminal of p53 was required for the interaction between p53 and RNA, which interfered with its DNA binding capacity [39]. Thus, we postulated that the C terminal of p53 determined the interaction of p53 and HOXB1. Further studies indicated that RBM47 could impair the interaction between HOXB-AS1 and p53, promoting p53 nuclear entry. Previous studies have reported that p53 nuclear import could activat p53 target genes [40, 41]. In our study, we found RBM47 and HOXB-AS1 could regulate the expression of p53 target genes, including p21, GADD45A, and GADD45B. Functional assays verified that RBM47 inhibited RCC progression through HOXB-AS1.

In conclusion, our study demonstrated that RBM47 was downregulated, which restrained RCC progression in RCC. RBM47 was regulated by H3K27ac in its promotor. RBM47 could inhibit the nuclear import of p53 through impairing the interaction between HOXB-AS1 and p53. Moreover, we observed that RBM47 had a synergistic anticancer effect with sunitinib both in vivo and in vitro.

## Methods and materials

### Patients and specimens

Human RCC tissues were collected at The Second Affiliated Hospital of Fujian Medical University (Quanzhou, China) from 2019 to 2022. The informed consent was obtained from all patients. This study was approved by the Ethical Committee of The Second Affiliated Hospital of Fujian Medical University.

### Cell lines

Human RCC cell lines (786-O, ACHN, 769-P, and Caki-1) and normal renal cell HK-2 cells were obtained from the Cell Bank Type Culture Collection of the Chinese Academy of Sciences (Shanghai, China). 786-O, ACHN, and 769-P cells were cultured in RPMI-1640 medium (Gibco, USA) containing 10% FBS (Gibco, USA). Caki-1 cells were cultured in McCoy’5a medium (Gibco, USA) supplemented with 10% FBS. All cells were cultivated at 37 °C with 5% CO_2_.

### RNA extraction and quantitative real-time PCR analysis

Trizol reagent (Invitrogen, USA) was used for total RNA extraction. mRNA was reverse transcribed to cDNA using the reverse transcription kit (Takara, Japan). Then, the mRNA was detected using SYBR Premix Ex TaqII Kit (TaKaRa, Japan). The relative mRNA expression was calculated using the 2^^-ΔΔCt^ method, and GAPDH was used as internal control. The detailed primer information was listed in Supplementary Table S1.

### Western blot assay

RIPA lysis buffer (Beyotime Biotechnology, China) was used to extract the protein. After SDS-PAGE, the proteins were transferred to the PVDF membranes. The membranes were blocked in the 5% BSA for an hour, and incubated with primary antibodies at 4 °C overnight. After washing for 5 times, the membranes were incubated with secondary antibodies and detected using the ECL detection system (Bio-Rad, USA). The detailed antibodies information was listed in Supplementary Table S2. The original western blot gels were shown in the supplementary materials.

### CCK-8 cell viability assay

The transfected RCC cells were seeded into 96-well plates with a density of 1000 cells/well. Then, 10% CCK-8 solution was added into each well and incubated for 2 h. Then, the absorbance was detected at 450 nm.

### Colony formation assay

The transfected RCC cells were seeded into 6-well plates with a density of 1000 cells/well. After 2 weeks, the cells were washed with PBS and fixed with 4% paraformaldehyde, followed by stained with 0.4% crystal violet. Then, the colonies were photographed.

### Wound healing assay

Upon the transfected RCC cells grow up to 90% confluence, 200 μL pipette tip was used for creating the wound. The wound was photographed at 0 h. After 24 h or 36 h, the wound was photographed again.

### Transwell migration assays

For transwell migration assays, 200 μL medium with 10% FBS containing 2 × 10^4^ transfected RCC cells was plated into the upper chambers, 650 μl serum-free medium was placed into the lower chamber. After 24 h, the chambers were fixed and stained with 0.4% crystal violet. Then, the images were photographed.

### Tumor xenograft model

Animal studies were approved by the Ethical Committee of The Second Affiliated Hospital of Fujian Medical University. A total of 1 × 10^6^ 769-P cells stably overexpressing RBM47 or vector were suspended in 100 uL matrigel and injected into the dorsal flank of 5-week-old nude mice. The tumor longest diameter (a) and widest vertical width (b) were measured every 4 days. The tumor volume = 0.5 × a × b^2^. Finally, after 5 weeks, the tumor tissues were surgical removed and weighted.

For animal model with sunitinib treatment, mice were orally treated with vehicle or sunitinib (40 mg/kg/day) for 3 weeks when the volume of xenograft reached 150 mm^3^.

### RNA pulldown assay

About 1 × 10^7^ RCC cells were lysed and incubated with biotin-labeled probes at RT for 30 min. Then, the streptavidin magnetic beads were added to incubate for another 30 min. After washed for 6 times using RNA pulldown washing buffer, the precipitated proteins were detected using western blot.

### RNA immunoprecipitation (RIP)

RNA immunoprecipitation assay was performed using Magna RIP^TM^ Kit (Millipore, USA). In brief, 2*10^7^ RCC cells were lysed and incubated with the indicated antibodies for 30 min at RT. Then, protein A/G magnetic beads were added into the antibodies/protein complex overnight at 4 °C. After washed for 3 times, the RNA was extracted and detected using qPCR.

### Immunofluorescence assay

The transfected RCC cells were seeded on the 24-well glass chamber slides. Then, the cells were fixed with 4% paraformaldehyde for 15 min and permeabilized with 0.25% Triton X-100 for 10 min. After washed with PBS 3 times, the cells were incubated with primary antibodies overnight at 4 °C, followed by incubating with secondary antibodies for an hour at RT. The nucleus was stained with DAPI. The images were observed by fluorescence microscope.

### RNA fluorescence in situ hybridization (FISH)

The transfected RCC cells were seeded on the 24-well glass chamber sliders. Then, the cells were fixed with 4% paraformaldehyde for 15 min and permeabilized with 0.25% Triton X-100 for 10 min. After washed with PBS 3 times, the cells were incubated with specific probes. The nucleus was stained with DAPI. The images were observed by fluorescence microscope.

### Chromatin immunoprecipitation (ChIP)

ChIP assay was performed using the Simple ChIP Enzymatic Chromatin IP Kit (CST, USA) according to the manufacturer’s instructions. The precipitated DNAs were detected using qPCR. The detailed primers information was listed in Supplementary Table S1.

### Statistical analysis

All statistical analysis were conducted using GraphPad Prism 7.0. The differences between two groups was analyzed using unpaired or paired Student’s *t*‐test. Data are presented as mean ± SD form in at least three independent experiments. *p < 0.05; **p < 0.01; ***p < 0.001.

### Supplementary information

Supplementry figure and figure legends

Supplementary table

Original western blot gel

## Data Availability

The datasets supporting the conclusions of this article are available from the corresponding author upon reasonable request.
