# Supplementary material for: RBM47 restrains renal cell carcinoma progression and chemoresistance through interacting with lncRNA HOXB-AS1
Source: Cell Death Discov. 2023 Sep 2;9:329. doi: 10.1038/s41420-023-01623-7 (PMC10475063; doi:10.1038/s41420-023-01623-7)
Supplement: Supplementary file 1 — Supplementry figure and figure legends [file 41420_2023_1623_MOESM1_ESM.docx]

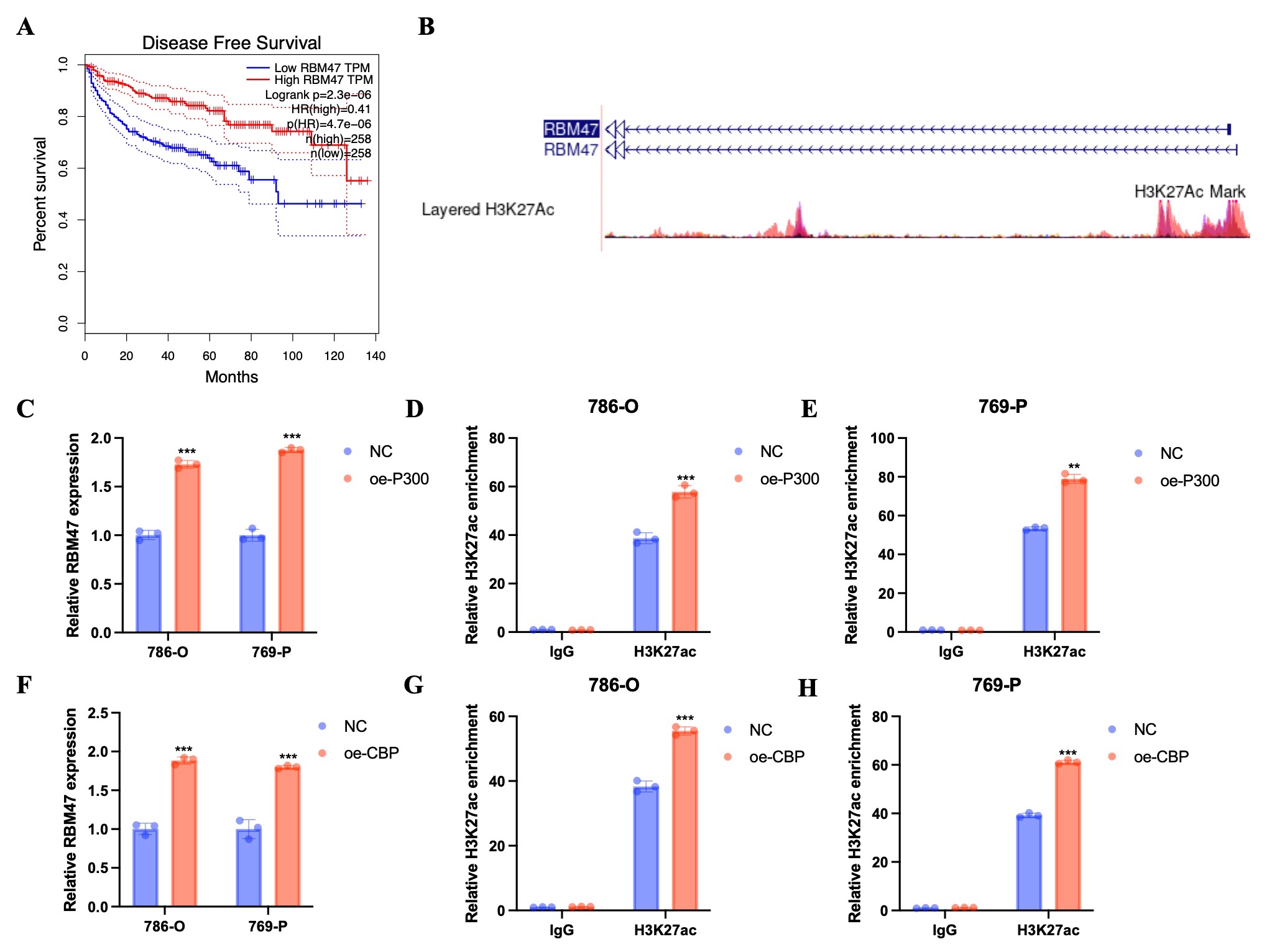


**Supplementary figure 1. A** Overall KIRC patients’ disease free survival determined by RBM47 expression using GEPIA2 platform. **B** The H3K27ac modification in RBM47 gene promotor using UCSC database. **C** Relative mRNA expression of RBM47 in RCC cells overexpression of P300. **D** and **E** Relative H3K27ac modification of RBM47 promotor in RCC cells overexpression of P300 using CHIP-qPCR. **F** Relative mRNA expression of RBM47 in RCC cells overexpression of CBP. **G** and **H** Relative H3K27ac modification of RBM47 promotor in RCC cells overexpression of CBP using CHIP-qPCR.


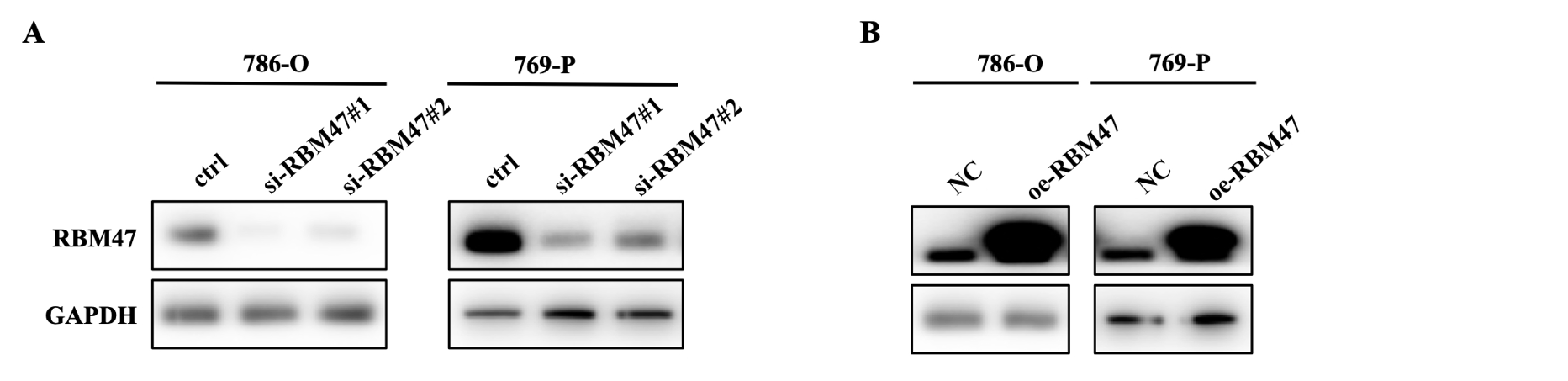


**Supplementary figure 2. A** Relative protein level of RBM47 measured by western blotting in RCC cells with RBM47 knockdown. **B** Relative protein level of RBM47 measured by western blotting in RCC cells with RBM47 overexpression.


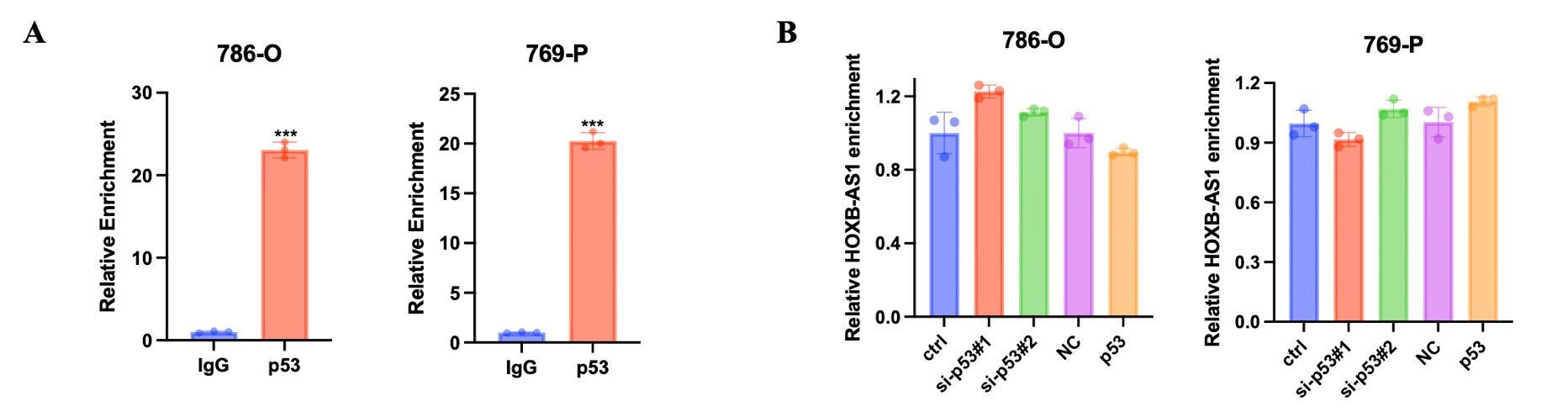


**Supplementary figure 3. A** RIP-qPCR assays showing the binding ability of HOXB-AS1 with p53 protein in RCC cell lines. **B** Relative mRNA expression of HOXB-AS1 in RCC cells with p53 knockdown or overexpression.


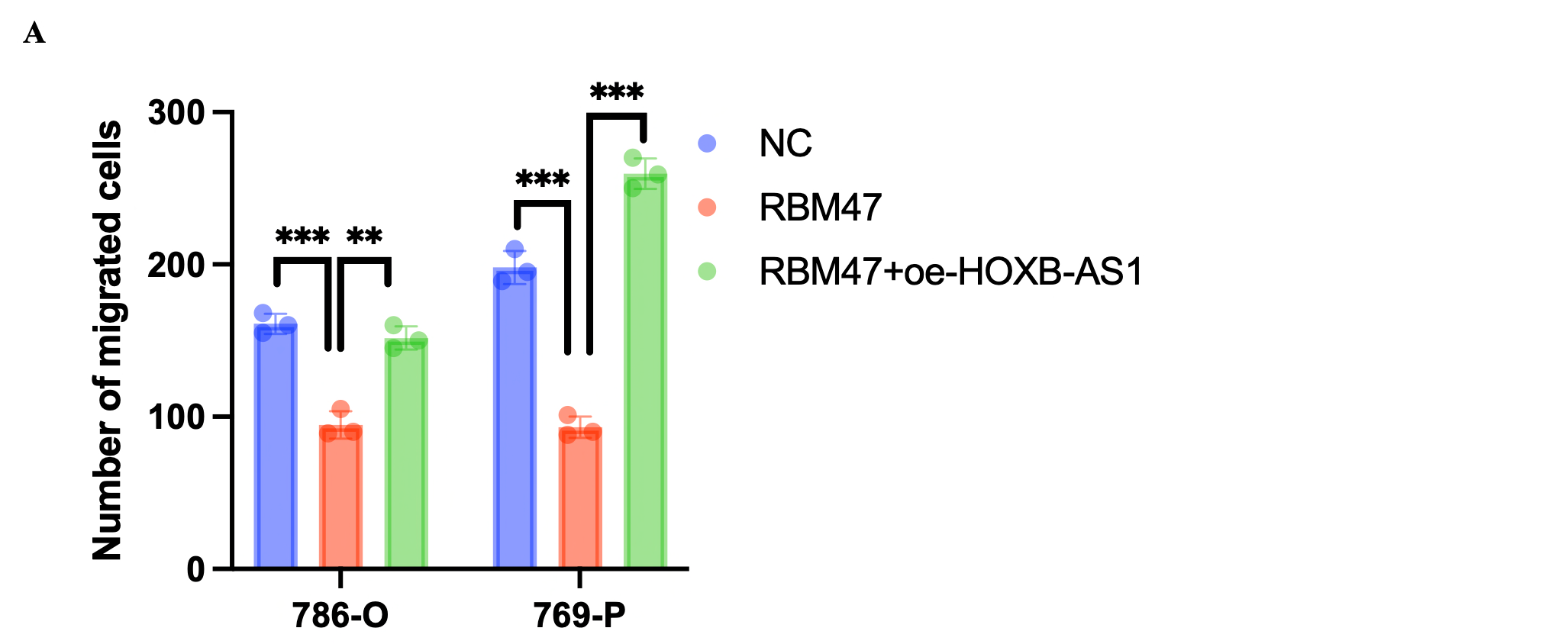


**Supplementary figure 4. A** Number of migrated cells in transwell assays using RBM47 overexpression with HOXB-AS1 overexpression or not.
