# Supplementary material for: RBM47 restrains renal cell carcinoma progression and chemoresistance through interacting with lncRNA HOXB-AS1
Source: Cell Death Discov. 2023 Sep 2;9:329. doi: 10.1038/s41420-023-01623-7 (PMC10475063; doi:10.1038/s41420-023-01623-7)
Supplement: Supplementary file 2 — Supplementary table [file 41420_2023_1623_MOESM2_ESM.docx]

**Supplementary table S1**. The primer sequences used for RT-PCR.

| Gene Names | Sequences (5’-3’) |
| --- | --- |
| RBM47-F | ATCAGCAATCCTTGGCTCAC |
| RBM47-R | CCTTGGGATTCCTCTGTTCA |
| HOXB-AS1-F | GGGGACTCCAGCGAAAT |
| HOXB-AS1-R | ACCCGAAGCCCAACCAC |
| TP53-F | CAGCACATGACGGAGGTTGT |
| TP53-F  rbm47-F | TCATCCAAATACTCCACACGC |
| GADD45A-F | GAGAGCAGAAGACCGAAAGGA |
| GADD45A-R | CACAACACCACGTTATCGGG |
| GADD45B-F | TACGAGTCGGCCAAGTTGATG |
| GADD45B-R | GGATGAGCGTGAAGTGGATTT |
| p21-F | TGTCCGTCAGAACCCATGC |
| p21-R | AAAGTCGAAGTTCCATCGCTC |
| GAPDH-F | GGAGCGAGATCCCTCCAAAAT |
| GAPDH-R | GGCTGTTGTCATACTTCTCATGG |

The primer sequences used for CHIP-qPCR

| Gene Names | Sequences (5’-3’) |
| --- | --- |
| RBM47-F | GTTCCCTTGCTTTCTGTC |
| RBM47-R | CCCACTGTGCTGCTTTAA |

**Supplementary table S2.** The antibodies information used in this study.

| Antibodies Names | Company | Catalog |
| --- | --- | --- |
| RBM47 | Abcam | ab167164 |
| GAPDH | Cell Signaling Technology | 92310SF |
| H3K27ac | Cell Signaling Technology | 8173S |
| TP53 | Cell Signaling Technology | 2527S |
| P21 | Cell Signaling Technology | 2947S |
| GADD45A | Cell Signaling Technology | 4632S |
| GADD45B | Abcam | ab230646 |
| Histone 3 | Cell Signaling Technology | 4499S |

**Supplementary table S3**. The siRNAs sequences used in this study.

| siRNAs Names | Sequences (5’-3’) |
| --- | --- |
| si-RBM47#1 | CACGGTGGCTCCAAACGTTCA |
| si-RBM47#2 | CGCTTGACTATTTATGAAGAATT |
| si-HOXB-AS1 | GGAAGAGGATTGTGAAGTTTA |
| si-CBP | CCGGCCCGATAACTTTGTGATGTTTCTCGAGAAAC AT C A C A A AGTTATCGGGTTTTT |
