# Supplementary material for: RBM47 restrains renal cell carcinoma progression and chemoresistance through interacting with lncRNA HOXB-AS1
Source: Cell Death Discov. 2023 Sep 2;9:329. doi: 10.1038/s41420-023-01623-7 (PMC10475063; doi:10.1038/s41420-023-01623-7)

Figure 1D

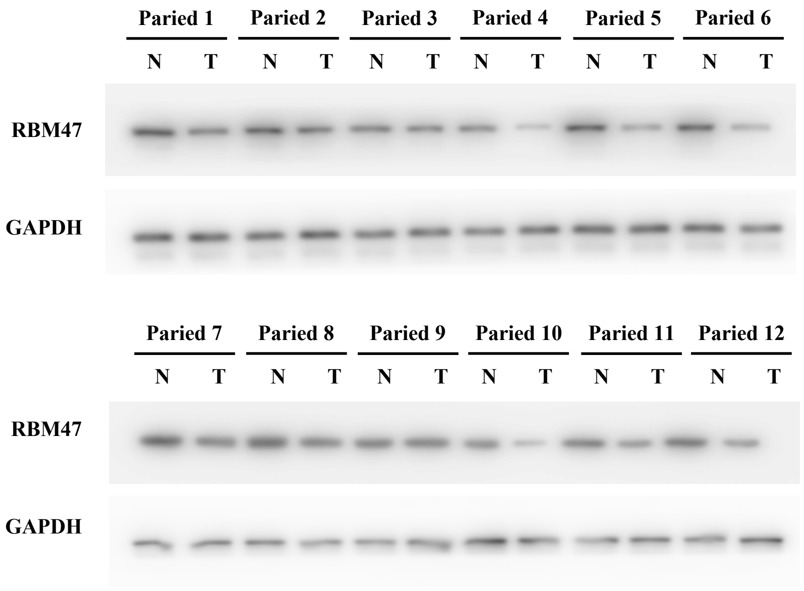

Figure 1F

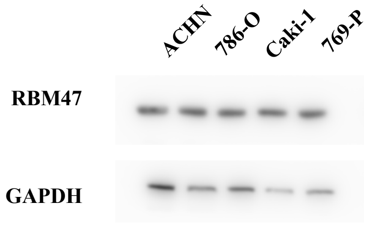

Figure 2E

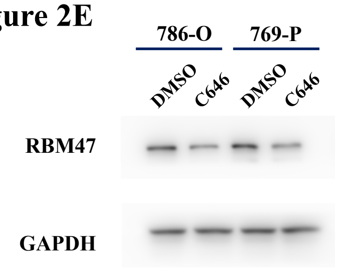

Figure 2H

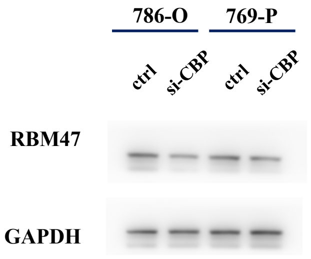

Figure 5C

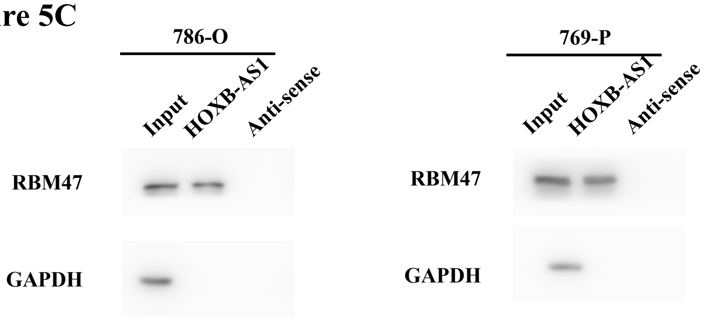

Figure 5E

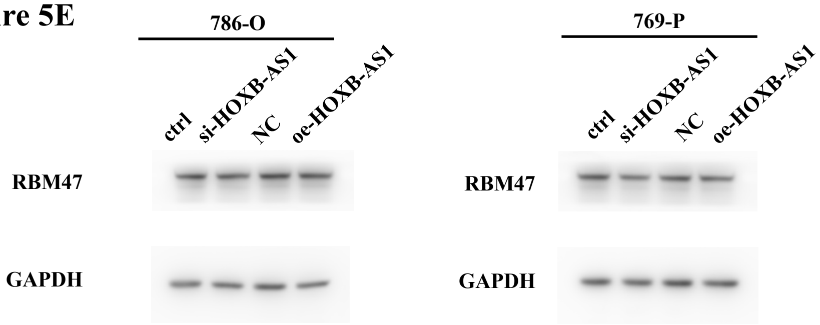

Figure 6B

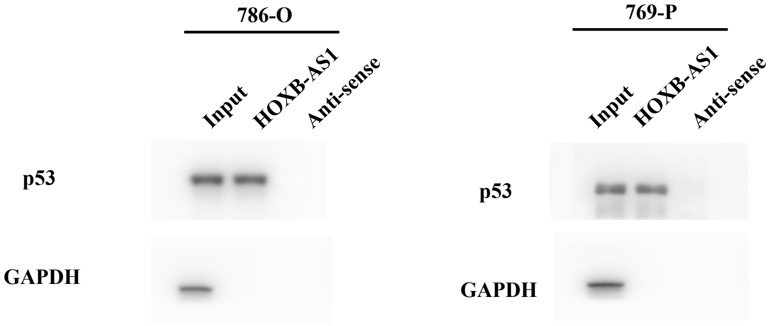

Figure 6C

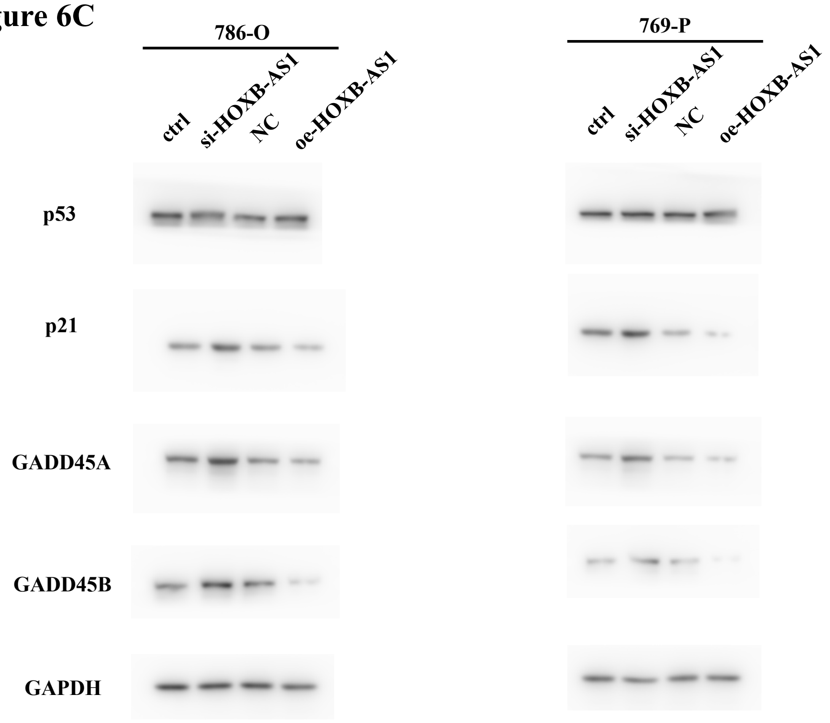

Figure 6E

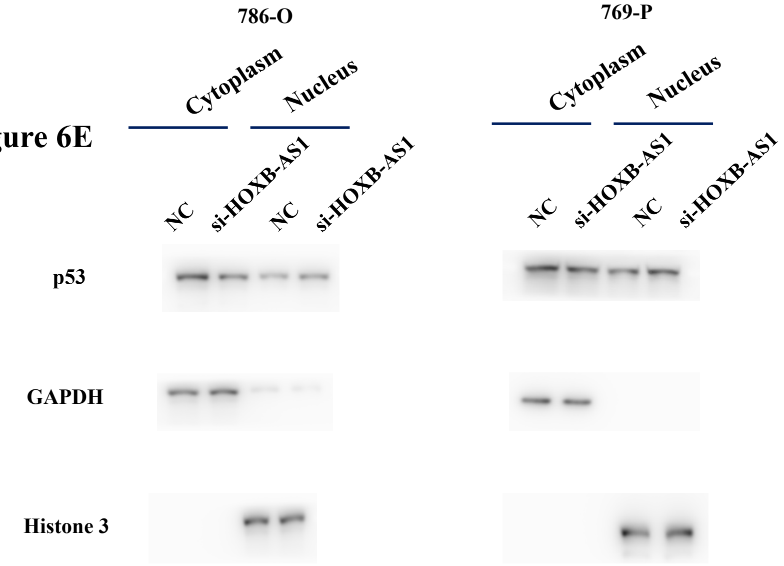

Figure 6F

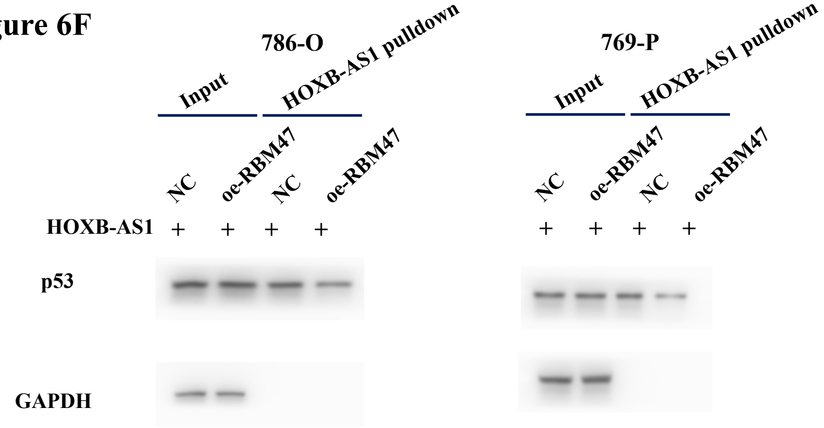

Figure 7B

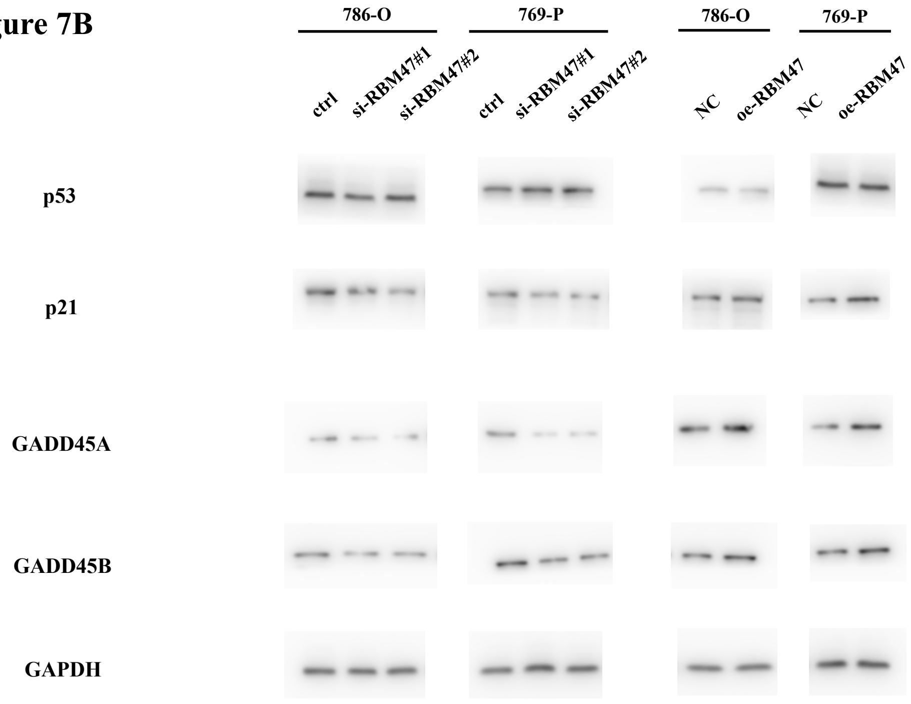

Figure 7F

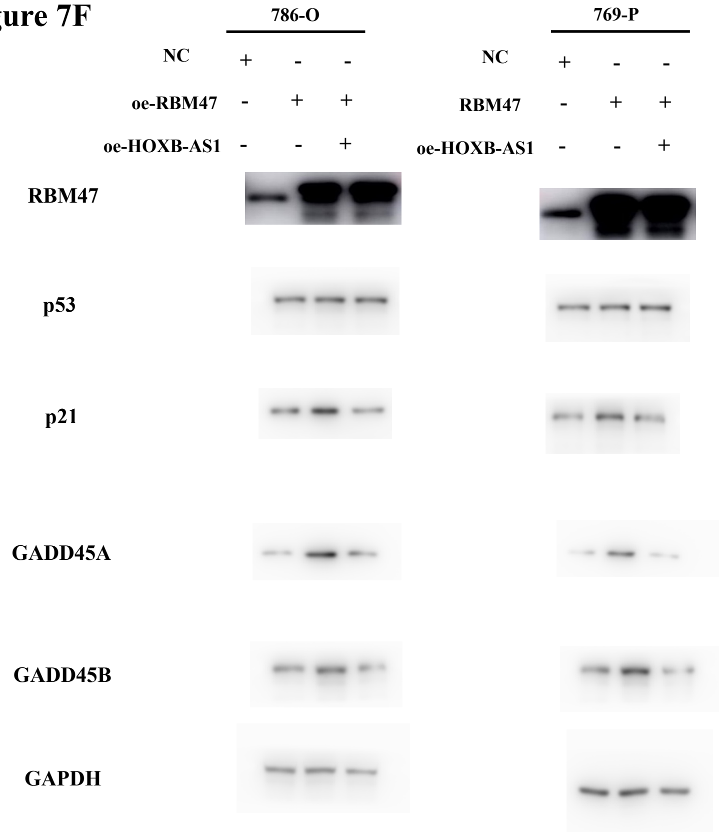

Supplementary Fig 2

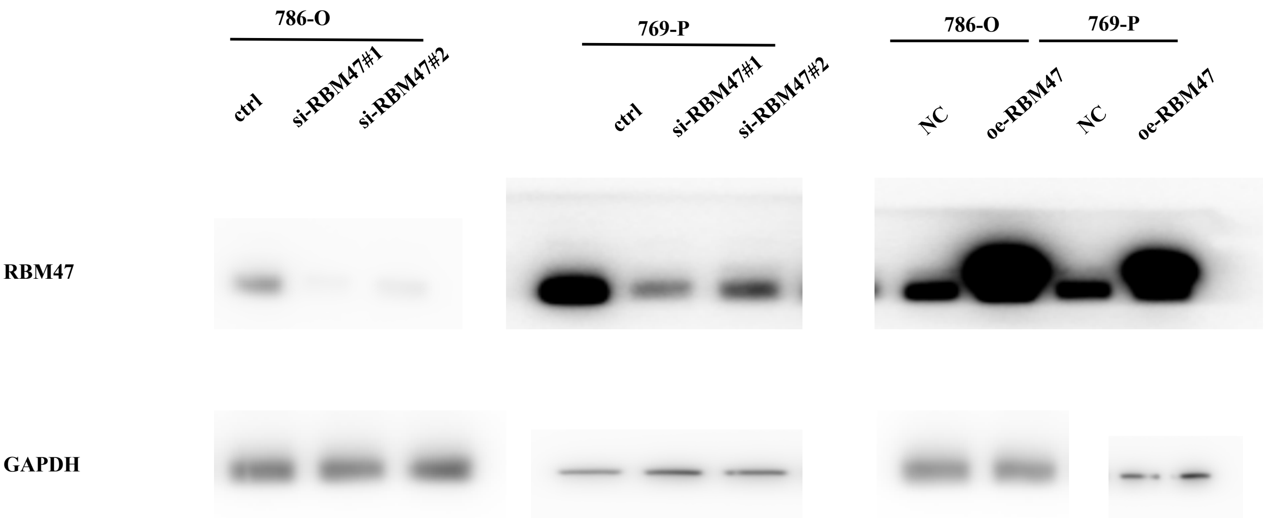

Supplement: Supplementary file 3 — Original western blot gel [file 41420_2023_1623_MOESM3_ESM.pdf]
